# Multi-omics reveals hypertrophy of adipose tissue and lipid metabolism disorder *via* mitochondria in young mice under real-ambient exposure to air pollution

**DOI:** 10.3389/fphar.2023.1122615

**Published:** 2023-03-23

**Authors:** Honglin Si, Tianlin Gao, Jing Yang, Jing Zhu, Ying Han, Chengwei Li, Jianxin Wang, Jianyu Li, Yanjie Zhao, Lei Chen, Yuxin Zheng, Menghui Jiang

**Affiliations:** ^1^ School of Public Health, Qingdao University, Qingdao, China; ^2^ School of Life Science and Technology, ShanghaiTech University, Shanghai, China; ^3^ Linyi Center for Disease Control and Prevention, Linyi, China

**Keywords:** PM2.5, young mice, adipose tissue, lipid metabolism, mitochondria, multiomics

## Abstract

Air pollution has become one of the most serious health risks as a result of industrialization, especially in developing countries. More attention has been drawn to the relationship between obesity/overweight and fine particulate matter (PM2.5). Especially for susceptible populations, the impact of air pollution on children and adolescents has attracted more public attentions. However, the detailed underlying mechanism influencing obesity or overweight under PM2.5 exposure is still unknown. Therefore, young mice were exposed to PM2.5 using the real-ambient exposure system that we previously established in Shijiazhuang city. Compared with the traditionally concentrated air particle (CAP) system, our real-ambient exposure system provides similar PM2.5 concentrations and characteristics as outdoor ambient air and minimizes the influence of external interfering factors. After 8 weeks of exposure to PM2.5, the weight of gonadal white adipose tissue (gWAT) and subcutaneous white adipose tissue (sWAT) was considerably increased, accompanied by a significantly enlarged size of adipocytes in sWAT. Importantly, multiomics analysis indicated altered metabolites involved in the lipid metabolism pathway, and transcriptomic analysis revealed notably changed signaling pathways related to fatty acid metabolism. Moreover, the mtDNA copy number, mitochondrial activity and fatty acid oxidation (FAO) were increased in the liver under PM2.5 exposure. Taken together, our research investigated the hypotrophy of adipose tissue in young mice, supported an imbalance in lipid metabolism based on multiomics analysis, and revealed disordered mitochondrial function under PM2.5 exposure. Our study provided new insight into the hazardous effects of air pollution, and extended our understanding on the underlying mechanism.

## 1 Introduction

Air pollution has become a global disease burden, contributing to 7.6% of the total annual mortality rate and 103.1 million deaths annually (Adar et al., 2014; Hassan Bhat et al., 2021). Recently, more and more studies revealed that air pollution contributed to the occurrence of obesity and overweight (Smith et al., 2020), especially in the pediatric population (Vrijheid et al., 2020). In fact, due to a faster rate of breathing, children can inhale more air pollutants than adults, which makes them one of the most susceptible populations to air pollution (Sly and Flack, 2008; Guo et al., 2019). A study showed that air pollution can cause disruptions of fatty acid metabolism in young people living near roads and may increase the risk of obesity-related metabolic diseases in adolescents (Chen et al., 2019). Accumulating epidemiological research has revealed that childhood exposure to air pollution contributes to overweight and a higher body mass index (BMI) in children (Tong et al., 2022). However, the underlying mechanism remains to be clarified. Therefore, in this study, young mice were used in our real-ambient exposure system to mimic the characteristics of childhood.

Overweight and obesity are characterized by the expansion of white adipose tissues (WATs), which occurs due to the storage of excessive lipid droplets, particularly in gonadal WAT (gWAT) and subcutaneous WAT (sWAT) (Scheja and Heeren, 2016). Obesity is usually accompanied by metabolic dysfunction, especially closely related to lipid metabolism. Additionally, the liver is a crucial organ in the regulation of lipid metabolism (Hellerstein, 1999; Wallace and Metallo, 2020). An increasing number of epidemiological studies have found that both short and long air pollution exposure contribute to an imbalance in lipid metabolism, which strongly facilitates the development of overweight and obesity (Kim et al., 2019; Furlong and Klimentidis, 2020; Simkova et al., 2020).

As the main site for the oxidation of fatty acids, mitochondria are crucial for maintaining the balance of lipid metabolism (Ipsen et al., 2018). Importantly, mitochondria are involved in the tricarboxylic acid cycle (TCA) and oxidative phosphorylation pathways. Accumulating studies have illustrated that air pollution contributes to mitochondrial dysfunction, mitochondrial metabolic abnormalities, and morphological abnormalities (Li et al., 2015a; Liu et al., 2020; Gao et al., 2022). It has been reported that the quantity and size of mitochondria are also changed under PM2.5 exposure (Mansouri et al., 2018).

The real-ambient exposure system that was previously established in Shijiazhuang city was applied in this study (Jiang et al., 2020; Ding et al., 2021). Compared to the concentrated ambient particle (CAP) exposure system, our system maintains the original chemical characteristics of PM2.5 and a concentration of PM2.5 similar to that of ambient PM2.5. In addition, our system maintains constant temperature, pressure, humidity, and low noise conditions without interrupting the normal activities and feeding of animals during the exposure period. Therefore, our real-ambient exposure system provides a perfect model for studying the harmful effects of PM2.5.

In our study, we examined the hypertrophy of adipose tissue in young mice, identified the disorder of lipid metabolism with multi-omics analysis and illustrated the increased mitochondrial activity in the real-ambient exposure system.

## 2 Materials and methods

### 2.1 Animals and whole-body inhaled PM exposure

Three-week-old female C57BL/6 mice were purchased in Beijing from Vital River Company and acclimated for 7 days after arrival. As per our previous protocol (Li et al., 2019; Jiang et al., 2020), the mice were randomly assigned to the filtered air (FA) and particulate matter (PM) groups in the real-ambient exposure system during winter in Shijiazhuang city (*n* = 10). Briefly, individually ventilated cages (IVCs) were linked directly to filtered air (FA group) or ambient outdoor air (PM group) after removing PM2.5 in ambient indoor air by three layers of high-efficiency particulate air (HEPA) filters. During the winter, the mice were exposed for 16 h per day (from 7:00 a.m. to 11:00 p.m.) for 4 or 8 weeks, and the mice were not restricted from eating or moving around. The experimental conditions were a 12-h daylight/12-h dark cycle. The room temperature was kept between 22 and 24°C. The experimental protocol was approved by the Animal Care and Use Committee of Qingdao University.

### 2.2 Tissue collection

After 4 or 8 weeks of PM2.5 exposure, inferior vena cava blood collection, and the serum was collected after centrifugation. The liver, gWAT and sWAT were collected and immediately fixed in 4% PFA or frozen at −80°C.

### 2.3 Pathological staining

The liver, gWAT and sWAT were fixed in 4% PFA, paraffin-embedded, sectioned, and stained with hematoxylin-eosin (H&E) to observe the histomorphology. Mitochondria-related genes, including *CPT1* (catalog #ab128568; Abcam), *CPT2* (catalog #ab181114; Abcam), *PGC1α* (catalog #2178s; Cell Signaling Technology, Danvers, MA) and *COX4* (catalog #11967; Cell Signaling Technology, Danvers, MA), were examined by immunohistochemistry and immunofluorescence. Briefly, the paraffin-embedded tissues were sectioned, deparaffinized with xylene, dehydrated with ethanol, blocked with 10% FBS, incubated with primary and secondary antibodies, and stained with DAB substrate solution. Finally, under a microscope, the color of the antibody staining in the tissue sections was observed.

### 2.4 RNA extraction and qPCR

TRIzol reagent was utilized to extract total RNA from liver, gWAT, and sWAT tissues according to the user’s manual. Reverse transcription experiments were performed to obtain cDNA templates according to the instructions (Accurate Biology, Hunan), and further real-time quantitative PCR experiments were carried out to ascertain the expression of target genes using the QuantStudio Q7 RT–PCR system. The 2^−ΔΔCT^ approach was used to calculate the target molecule’s expression level relative to β-actin. In Supplementary Table 1, the primer sequences applied in the studies are listed.

### 2.5 Lipid content

According to the user’s manual, the liver was homogenized in ethanol, and lipids were extracted from the supernatant after centrifugation. TG, TC and FFA levels in the liver and serum were measured using Nanjing Jiancheng kits, and VLDL levels in the liver and serum were measured using enzyme-linked immunosorbent assay (ELISA) kits (Fankew, Shanghai). After incubation for the indicated time, the absorbance was detected by a microplate spectrophotometer, and the concentration was calculated based on a standard curve.

### 2.6 Metabolomic analysis

The metabolites in serum and liver were analyzed by extraction, computer detection and statistical analysis. First, 25 mg liver was added to 500 μL of extract solution (methanol:acetonitrile:water = 2:2:1) and 100 μL of serum sample was added to 400 μL of extract solution (methanol:acetonitrile = 1:1), both of which contained the isotopically labeled internal standard mixture. Mix well and sonicate for 10 min under ice-water bath conditions, noting that the liver needs to be processed by grinding. The supernatant solution was obtained by centrifugation at 12,000 rpm for 15 min at 4°C after 1 hour at −40°C. The target compounds were chromatographically separated on a Waters ACQUITY UPLC BEH Amide (2.1 mm × 100 mm, 1.7 μm) column using a Vanquish (Thermo Fisher Scientific) ultra-performance liquid chromatograph. The A-phase of liquid chromatography was aqueous containing 25 mmol/L ammonium acetate and 25 mmol/L ammonia, and the B-phase was acetonitrile. The data collection was performed on a QE HFX mass spectrometer (Orbitrap MS, Thermo) under the control of acquisition software (Xcalibur, Thermo). The raw data were also processed using ProteoWizard software.

### 2.7 Lipidomics analysis

The adipose tissue was ground, and water and methanol solution were added to the homogenate at a ratio of 2:1:4 to extract lipid components. To enable subsequent measurement, 250 mL of methyl tert-butyl ether (MTBE) comprising lipid standards was added after vortex mixing. The samples were bathed in 10°C water for 30 min (ultrasonic frequency 40 kHz, power 100 W). The organic phase was then separated by centrifugation at 10°C (1,400 × g) for 10 min. LC-MS analysis was performed using ultra-high pressure liquid chromatography combined with a Sciex Triple TOF 6600 liquid chromatograph. The Thermo Hypersil Gold C18 column (100 mm × 2.1 mm, 1.9 μm) was used. The mobile phase A was water-acetonitrile (6:4 v/v, containing 0.1% formic acid and 10 mmol ammonium formate), and the mobile phase B was acetonitrile-isopropanol (1:9 v/v, containing 0.1% formic acid and 1 mmol ammonium formate). Data analysis was performed using Peak view software to analyze lipid composition.

### 2.8 Transcriptome analysis

Total RNA was extracted from the liver, and the quality of the RNA was examined for concentration and degradation. mRNA was enriched with oligo-(dT) beads, and subsequently, cDNA was generated with poly (A) added to the 3′ end. After purification, sequencing adapters were added to the QiaQuick PCR kit, agarose gel electrophoresis was used to assess the size of the fragments, and PCR was performed to amplify the cDNA. After library construction, the quality inspection of the library was qualified, and PE150 sequencing was performed using the Illumina NovaSeq platform. KOBAS software was used to perform a KEGG enrichment analysis.

### 2.9 Statistical analysis

GraphPad Prism 8.3.0 software was used for the statistical analysis of the experimental data, and the unpaired two-tailed *t*-test was run. Error bars represent ± SEM, and statistical significance was considered when the *p* value was less than 0.05. *, *p* < 0.05, **, *p* < 0.01, and ***, *p* < 0.001. Regarding metabolomics analysis, after preprocessing the data, a set of observed potentially correlated variables was transformed into linearly uncorrelated variables by orthogonal transformation using principal component analysis (PCA). Using SIMCA software (V16.0.2, Sartorius Stedim Data Analytics AB, Umea, Sweden), the data were formatted by logarithmic (LOG) transformation plus centralization (CTR), and then automatic modeling analysis was performed to obtain scores indicating the first and second ranked principal components, respectively [PC (1) and PC (2)]. The orthogonal partial least squares-discriminant analysis (OPLS-DA) statistical method was also used to filter out orthogonal variables in metabolites that were not correlated with categorical variables, and non-orthogonal and orthogonal variables were analyzed separately. Thus, a more reliable letter of correlation between group differences of metabolites and experimental groups was obtained. Finally, SIMCA software was used to obtain the Variable Importance in the Projection (VIP) by logarithmic (LOG) transformation plus UV formatting of the data. VIP is the importance of the variables to the model, which describes the overall contribution of each variable to the model, and is usually set at a threshold value of VIP > 1, *p* value < 0.05.

## 3 Results

### 3.1 Chronic real-ambient PM2.5 exposure facilitated the hypertrophy of white adipose tissue

**FIGURE 1:**
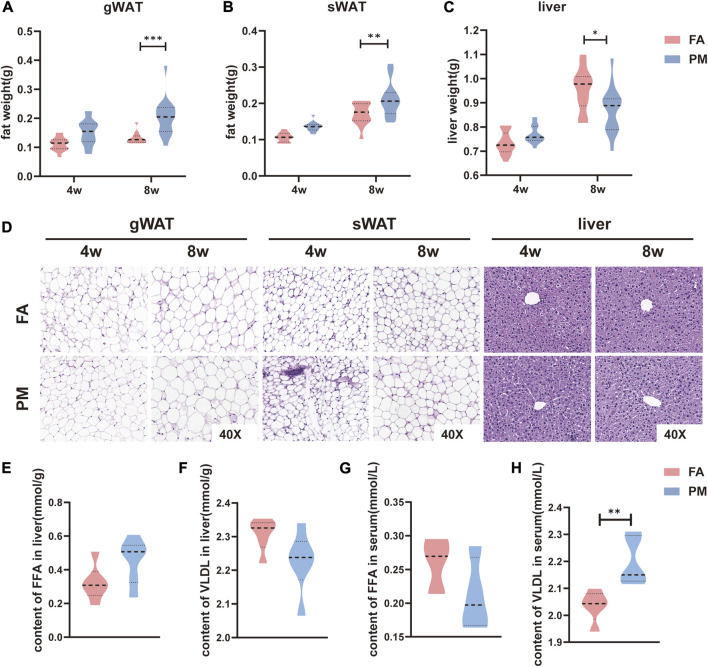
Hypertrophy of adipose tissue overgrowth in young mice after PM2.5 exposure **(A-C)** The mass of gWAT, sWAT and liver tissue after exposure to PM2.5 for 4 and 8 weeks (*n* = 17–20). **(D)** Hematoxylin and eosin (H&E) staining was used to observe the morphology of adipose tissue and liver tissue (*n* = 4). **(E-F)** The contents of FFAs and VLDL in the liver after exposure for 8 weeks (*n* = 5). **(G-H)** Serum FFA and VLDL contents after exposure for 8 weeks (*n* = 5). *, *p* < 0.05; **, *p* < 0.01; ***, *p* < 0.001.

To examine the adverse effect of PM2.5 on adipose tissue, young mice were exposed to the real-ambient exposure system in Shijiazhuang during the winter of 2019. During the exposure period, the daily average ambient PM2.5 concentration reached 140.8 μg/m^3^ (Ji et al., 2022). After exposure for 4 or 8 weeks, the sWAT and gWAT were isolated and weighed. Interestingly, after 8 weeks of exposure, the gWAT and sWAT mass increased significantly in the PM group compared with the FA group (Figures 1A, B). Considering the close crosstalk between liver and adipose tissue (Scheja and Heeren, 2016), the liver weight was evaluated as well. Conversely, the liver weight was markedly reduced in the PM group compared with the FA group (Figure 1C). Because of the contribution of hypertrophic adipocytes to the growth of adipose tissue (Ghaben and Scherer, 2019), hematoxylin-eosin (H&E) staining was used to determine the adipocyte size in sWAT and gWAT. Consistently, after exposure for 8 weeks, the adipocytes of the sWAT in the PM group were considerably larger than those in the FA group, accompanied by a slightly increased adipocyte size in the gWAT in the PM groups (Figure 1D). Furthermore, the histomorphology of the liver was detected with H&E staining. However, the morphology was similar in the FA and PM groups (Figure 1D). Considering the pivotal role of fatty acids during the crosstalk of adipose tissue and liver (Fuchs et al., 2022), the levels of free fatty acids (FFAs) and very-low-density lipoproteins (VLDLs) in the liver and serum were measured by ELISA. We found a trend of higher levels of FFAs in the liver and lower levels in the serum in the PM group (Figure 1E, G). Importantly, the level of serum VLDL was strikingly increased in the PM group, accompanied by a decreasing trend in the liver, indicating the influx of VLDLs from hepatocytes to serum (Figure 1F, H). Taken together, our findings showed that chronic exposure to PM2.5 exacerbated the expansion of fat tissue.

### 3.2 Lipidomic remodeling of WAT and metabolomic reprogramming of serum and liver to PM2.5 exposure

**FIGURE 2:**
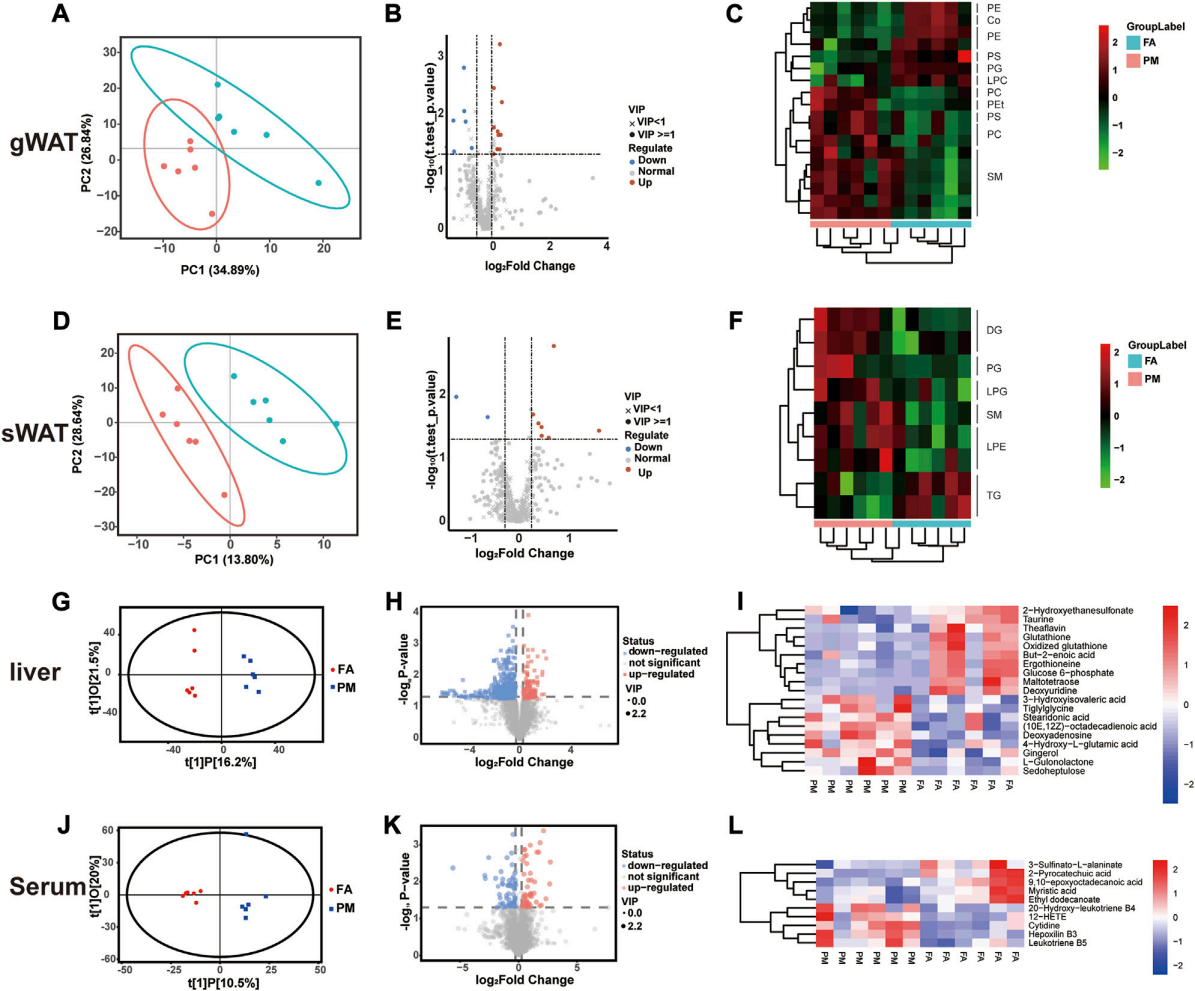
Metabolic reprogramming in gWAT, sWAT, liver and serum based on multiomics analysis **(A–F)** The lipidomic analysis results of gWAT and sWAT (*n* = 6), including PLS-DA plots **(A, D)**, volcano plots **(B, E)**, and cluster heatmaps **(C, F)**. **(G–H)** Liver and serum metabolomic analysis results (*n* = 6), including OPLS-DA plots **(G, J)**, volcano plots **(H, K),** and cluster heatmaps **(I, L).** PC [1] and PC [2] in partial least squares discriminant analysis (PLS-DA) denote the scores of the first and second ranked principal components, respectively. t[1]P in orthogonal partial least squares discriminant analysis (OPLS-DA) denotes the prediction score of the first principal component, indicating the difference between sample groups; t[1]O denotes the score of the orthogonal principal component, indicating the difference within sample groups.

It is generally known that lipid metabolism regulates the growth of adipose tissue (Samuel and Shulman, 2016). After exposure for 8 weeks, lipidomic analysis was performed in gWAT and sWAT. Based on PLS-DA, the lipid signature of gWAT and sWAT in the PM group was considerably distinct from that of the FA group (Figures 2A, D). According to volcano plots, some notably changed lipid species were observed in gWAT and sWAT (Figures 2B, E). To further identify the specific lipid species that were affected by PM2.5, all significantly altered lipid species were visualized by heatmaps. A total of 18 lipid species markedly changed in gWAT (Figure 2C), while nine species considerably changed in sWAT (Figure 2E). The lipid classes included diacylglycerol (DG), triacylglycerol (TG), acyl-carnitine coenzyme Q (CoQ), phosphatidylcholine (PC), phosphatidylethanolamine (PE), phosphatidylglycerol (PG), phosphatidylserine (PS), sphingomyelin (SM), phosphatidylethanol (PET), lyso-phosphatidylethanolamine (LPE), lyso-phosphatidylcholine (LPC) and lyso-phosphatidylglycerol (LPG) (Figure 2F).

The liver plays a crucial role in the expansion of adipose tissue by regulating lipid influx, and metabolites usually circulate in the bloodstream to realize crosstalk among tissues (Scheja and Heeren, 2016). Therefore, the metabolites in the liver and serum were examined with metabolomics analysis. Consistently, based on OPLS-DA, the metabolomic profiling of liver and serum in the PM group was notably distinct from that in the FA group (R^2^X = 0.36, R^2^Y = 0.989, Q^2^ = 0.211 in Figure 2G, R^2^X = 0.407, R^2^Y = 0.882, Q^2^ = 0.0422 in Figure 2J) (Figures 2G, J). Based on volcano and clustering heatmaps, when compared to the FA group, various metabolites in the liver and serum displayed significant differences in the PM group (Figures 2H, K). Consistently, the level of TG in the liver was increased after exposure to PM2.5 (Figure 2I). Glutathione is believed to be a key regulator in fat burning and storage (Rom et al., 2020). Glutathione was altered in the liver during PM2.5 exposure, accompanied by significant downregulation of the unsaturated fatty acid but-2-enoic acid (Figure 2I). At the same time, we also found that PM2.5 exposure had an effect on glucose metabolism, and its key metabolite glucose 6-phosphate was significantly reduced at the end of the exposure (Figure 2I). 12-Hydroxyeicosatetraenoic acid (12-HETE) and leukotriene B5 are considered inflammation markers (Maaløe et al., 2011; Cheng et al., 2019). Both of these metabolites notably increased in serum after PM exposure (Figure 2L). Taken together, these results revealed glycolipid metabolic remodeling in adipose tissue and liver when exposed to PM2.5.

### 3.3 Altered metabolism-related signaling pathways based on transcriptome analysis during PM2.5 exposure

**FIGURE 3:**
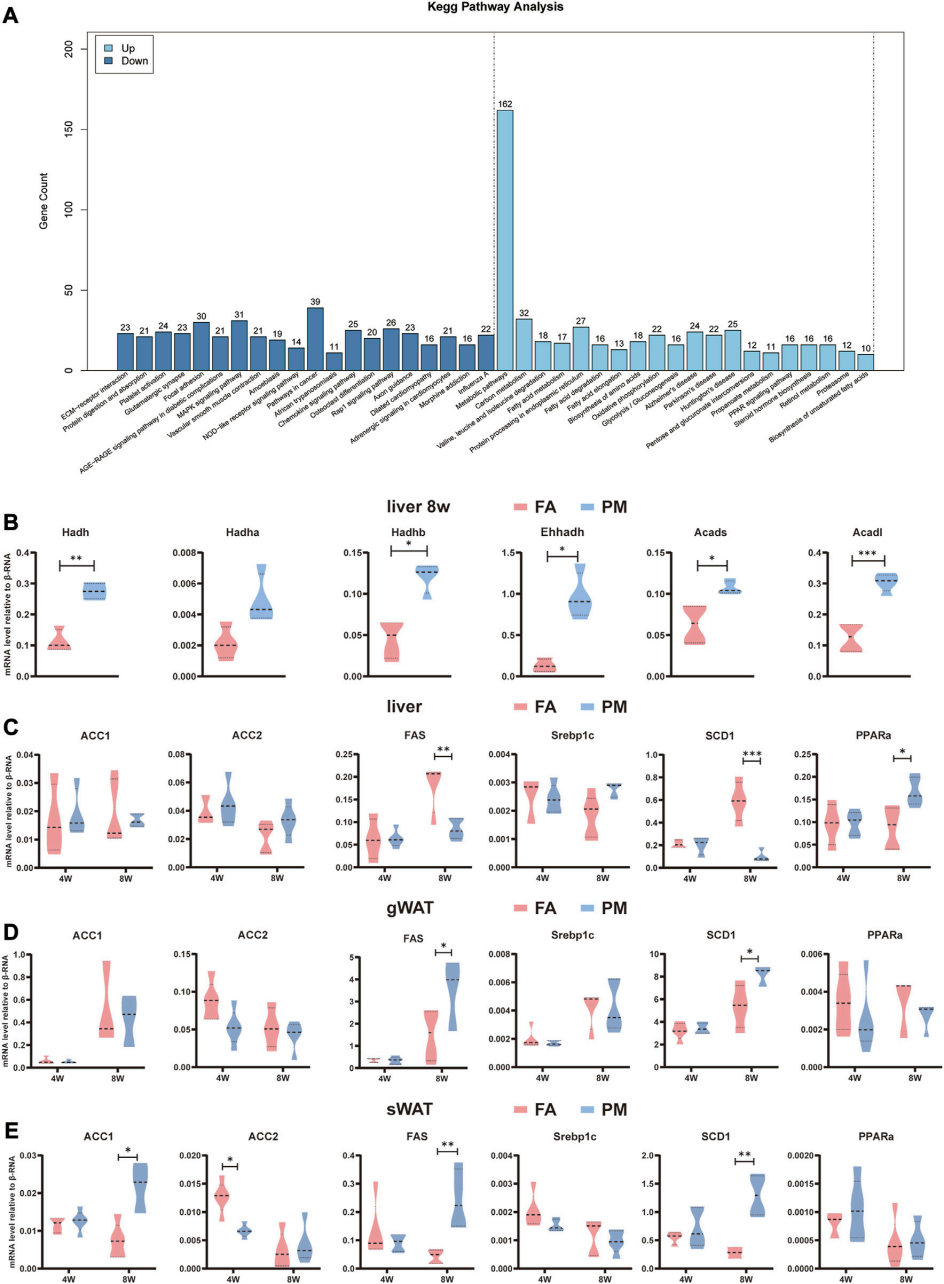
Altered metabolism-related signaling pathways based on transcriptome analysis **(A)** KEGG enrichment analysis according to transcriptome analysis in the liver after exposure for 8 weeks. Differential genes were screened according to *p* value < 0.05 and |log_2_FC|>12. The vertical axis indicates the number of differentially expressed genes, while the horizontal axis reflects the enrichment of the top 20 pathways that were significantly upregulated or downregulated (*n* = 4). **(B–D)** Relative mRNA levels of genes related to fatty acid synthesis and fatty acid oxidation in the liver, sWAT, and gWAT (*n* = 5). **(E)** Relative mRNA levels of β-oxidation-related genes in the liver (*n* = 5). *, *p* < 0.05; **, *p* < 0.01; ***, *p* < 0.001.

To investigate the underlying molecular mechanism, the liver was subjected to transcriptomic analysis. KEGG enrichment analysis revealed the top 20 upregulated and downregulated signaling pathways in the liver. Intriguingly, the notably changed signaling pathways included metabolic pathways, fatty acid metabolism, fatty acid degradation, fatty acid elongation, biosynthesis of unsaturated fatty acids, oxidative phosphorylation (Figure 3A). These pathways are well known to be involved in regulating lipid metabolic homeostasis. Besides, in agreement with the results of the metabolomic analysis of this study, glycolysis/gluconeogenesis, and pentose and glucuronate interconversion pathways were remarkably altered (Figure 3A).

Acetyl-CoA carboxylase 1 (*ACC1*), acetyl-CoA carboxylase 2 (*ACC2*), fatty acid synthetase (*FAS*), sterol regulatory element-binding transcription factor 1 (Srebp1c), and stearoyl-coenzyme A desaturase 1 (*SCD1*) participate in the *de novo* lipogenesis (DNL) pathway. Peroxisome proliferator-activated receptor alpha (*PPARα*) plays a key role in lipid oxidation. Compared to the FA group, the mRNA levels of *FAS* and *SCD1* were dramatically reduced (Figure 3B), whereas the mRNA levels of *PPARα* significantly increased in PM group after exposed for 8w (Figure 3C). Hydroxy acyl-CoA dehydrogenase (*Hadh*), acyl-CoA dehydrogenase (*Acad*), enoyl-CoA hydratase and 3-hydroxy acyl CoA dehydrogenase (*Ehhadh*) and cytochrome P450, family 4, subfamily a (*Cyp4a*) are involved in lipid oxidation (Hoek-van den Hil et al., 2013; Ipsen et al., 2018). These genes were found altered by transcriptome analysis, and were further validated with qPCR. Consistently, *Hadh*, *Hadhb*, *Ehhadh*, Acad long chain (*Acadl*) and Acad short chain (*Acads*) were greatly enhanced in the liver in the PM group (Figure 3C). In addition, the expression of metabolism-related genes was examined in gWAT and sWAT. The findings revealed that after exposure to PM2.5, the levels of *FAS* and *SCD1* were considerably increased in gWAT and sWAT, accompanied by higher levels of *ACC1* in sWAT (Figures 3D, E). These results suggested that PM2.5 exposure disturbed signaling pathways involved in metabolism in the liver and adipose tissue.

### 3.4 Increased mitochondrial activity under PM2.5 exposure

**FIGURE 4:**
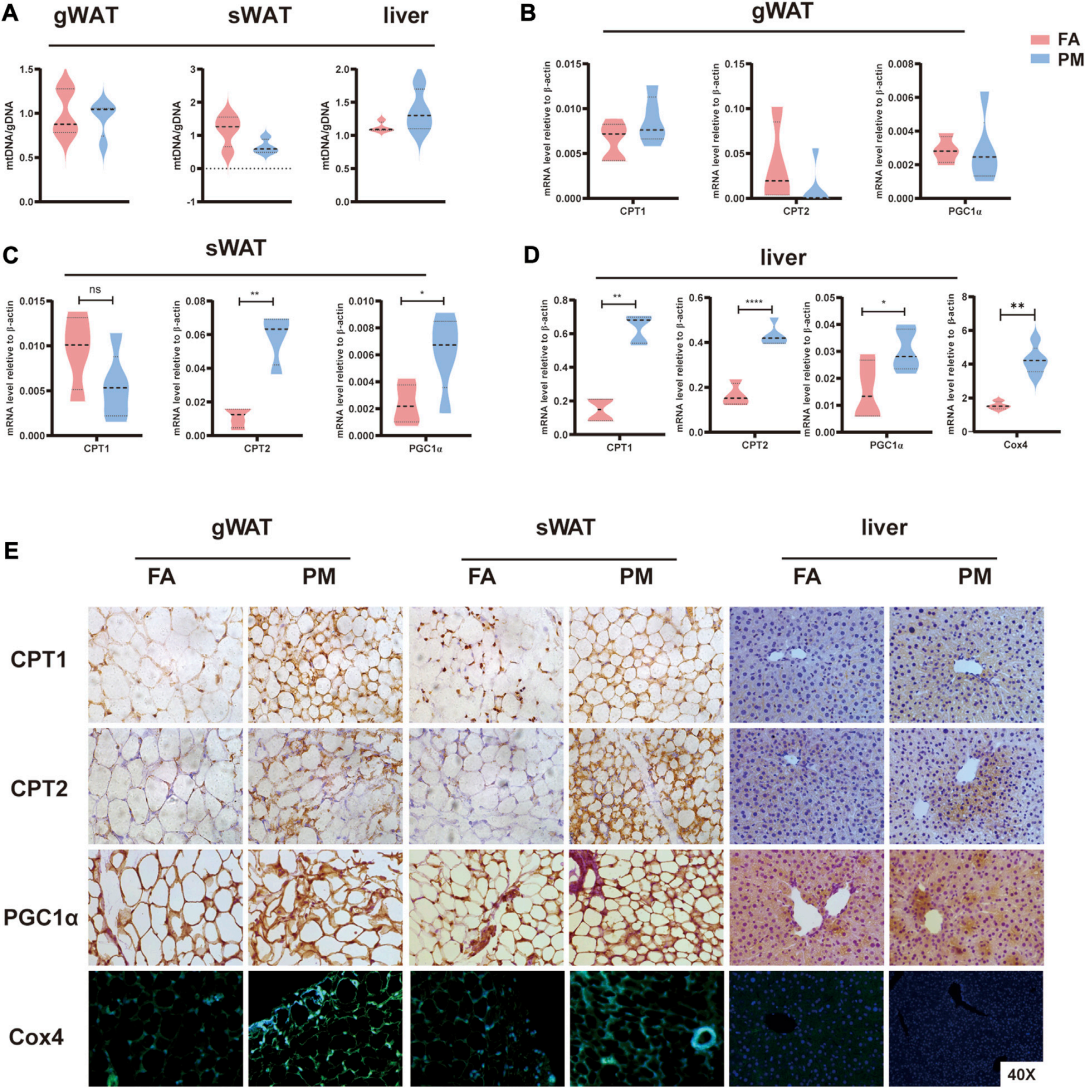
Abnormal mitochondrial activity in liver and adipose tissue under PM2.5 exposure **(A)** mtDNA copy number levels in gWAT, sWAT and liver tissue (*n* = 5). **(B–D)** The mRNA levels of the *CPT1*, *CPT2*, and *PGC1α* enzymes in gWAT, sWAT and liver tissue, respectively (*n* = 5). **(E)** Immunohistochemistry and immunofluorescence techniques were used to detect *CPT1*, *CPT2*, and *PGC1α* protein levels and mitochondrial numbers (*Cox4* labeling) in gWAT, sWAT and liver tissue (*n* = 3). *, *p* < 0.05; **, *p* < 0.01; ***, *p* < 0.001.

The primary function of mitochondria is maintaining energy homeostasis by breaking down lipids and other molecules (Thorp, 2019). Therefore, mitochondrial activity was evaluated after PM2.5 exposure. After 8 weeks of exposure, the mtDNA copy number were comparative in FA and PM groups in sWAT, gWAT and liver based on qPCR assays (Figure 4A). Carnitine palmitoyltransferase (*CPT1*, *CPT2*), as a mitochondrial enzyme, is critical in fatty acid oxidation (FAO) (Nassir and Ibdah, 2014). Peroxisome proliferator-activated receptor-gamma coactivator 1α (*PGC1α*) is considered a primary regulator of mitochondrial biosynthesis (Li et al., 2016; Jamwal et al., 2021). The levels of *CPT1*, *CPT2*, and *PGC1α* were investigated using qPCR and IHC staining in liver, gWAT, and sWAT. The levels of *CPT2* and *PGC1α* were considerably increased in sWAT, and the mRNA expression of *CPT1*, *CPT2* and *PGC1α* was considerably increased in the liver (Figures 4B–D). Consistently, the protein levels of these genes markedly increased in the gWAT, sWAT and liver after exposure for 8 weeks (Figure 4E). As a marker of mitochondria, cytochrome c oxidase subunit 4 (*Cox4*) was determined by immunofluorescence labeling. We found increased expression of *Cox4* in gWAT and sWAT after exposure to PM2.5 (Figure 4E). In addition, the mRNA expression of *Cox4* was significantly elevated in the liver after exposed to PM2.5 for 8 weeks (Figure 4D). These findings revealed that the number and activity of mitochondria increased during PM2.5 exposure.

## 4 Discussion

Air pollution has been considered as a worldwide public health concern and has drawn more attention recently (Ostro et al., 2018). As a primary component in air pollution, PM2.5 directly enters the blood and contributes to multiorgan toxicities (Goldberg and Villeneuve, 2008; Xu et al., 2016; Xu et al., 2018). Recently, accumulating research has shown that exposure to PM2.5 can act as an independent factor to disrupt the balance of lipid metabolism (McGuinn et al., 2019; Zhang et al., 2021). Air pollution can increase the BMI of children and can increase the probability of developing obesity, as reported by most epidemiological studies (Tong et al., 2022). The underlying molecular mechanisms, however, are not completely understood. Consistently, in our research, PM2.5 inhalation exposure caused remarkable hypertrophy of adipose tissue, accompanied by disturbances in lipid metabolism in young mice.

The PM2.5 concentration in Shijiazhuang is extremely high, with annual average PM2.5 concentrations as high as 138.8 μg/m^3^ in 2014–2016 (Xie et al., 2019). Therefore, our real-ambient exposure system was built in Shijiazhuang city, as described in our previous reports (Li et al., 2019). Simulating real environmental exposure is one of the innovative points of this paper. Our real-ambient exposure system built in Shijiazhuang provided flawless control with filtered air in which the PM was undetectable and air that maintained a similar and changeable PM concentration in the exposure chamber coinciding with the outdoor air environment. Moreover, in the real-ambient system, the characteristics of PM were comparable with those of outdoor air pollution. The composition of PM2.5 in our system was already measured in our previous study, with PAHs, PCBs and polychlorinated dibenzo-p-dioxins (PCDD) in organic extracts and metals and trace elements (Zn, Mn, As, Pb, Li, Cr, NO_3_^−^ and SO_4_^2−^) in the water-soluble fraction (Li et al., 2020). In addition, our system also maintains constant temperature, pressure, humidity and low noise levels, minimizing the effects on animal activity. Our system provides a perfect model for studying whole-body inhalation exposure to air pollution. In fact, we have already reported the toxicities of PM2.5 on the lung and cardiovascular system by applying the real-ambient exposure system (Cui et al., 2020; Su et al., 2020; Zhang et al., 2022).

According to a World Health Organization survey in 2020, 38.9 million children under the age of five were overweight worldwide, and the number of adolescents and adults population suffering from obesity reached 340 million and 650 million, respectively (Rajagopalan et al., 2018). A nationwide survey of obesity and overweight prevalence based on Chinese standards found overweight rates of 6.8% and 11.1% in children (<6 years) and adolescents (6–17 years), respectively, and obesity rates of 3.6% and 7.9% (Pan et al., 2021). Obesity in early childhood not only substantially increases the chances of obesity in adulthood but also causes sequelae, such as premature death, type 2 diabetes and cardiovascular disease. Recently accumulating epidemiological studies have supported the link between air pollution and obesity (Dong et al., 2014; Li et al., 2015b; Rohde and Muller, 2015). For instance, prenatal exposure to air pollution or childhood exposure to traffic pollution increases BMI and contributes to the development of obesity (Fioravanti et al., 2018; Tong et al., 2022). Consistently, our results show that whole-body PM2.5 exposure leads to significant hypertrophy of white adipose tissue in young mice, including sWAT and gWAT.

It is well known that the development of overweight and obesity is accompanied by excessive storage of lipids in adipose tissue (Park et al., 2021). Lipid metabolism was reported to be influenced by PM2.5 exposure, thereby affecting the expansion of adipose tissue (Zou, 2010; Chen et al., 2012; Pei et al., 2013). Lipidomics enables the systematic analysis of overall lipids, which in turn can accurately identify key lipid biomarkers in metabolic regulation and ultimately uncover the mechanisms of lipid action in a variety of life activities (Brügger, 2014). Based on lipidomics, the lipid metabolism was found disturbed in white adipose tissue after exposed to PM2.5 for 8 weeks. FFAs in tissues are stored and transported in the form of DG and TG and provide energy to the body (Alves-Bezerra and Cohen, 2017). Through lipidomics, our study found that TG was significantly reduced in sWAT after 8 weeks of PM2.5 exposure, but DG, a precursor of TG, was significantly increased. In addition, PC (a precursor for cholesterol synthesis, which turns excess lipids into smaller droplets for excretion) (Cole et al., 2011) and SM (which regulates cell membrane fluidity and cell transduction) (Hori et al., 2021) were significantly increased in gWAT.

The crosstalk between the liver and adipose tissue is pivotal for regulating lipid metabolism. For example, FFAs are released into the blood by WAT, enter and provide rapid mobilization fuel for the liver and ATP for gluconeogenesis and are esterified into more complex molecules, such as VLDL, in the liver (Scheja and Heeren, 2016). In addition to lipid metabolism, the liver plays a crucial role in the metabolism of carbohydrates, proteins, and amino acids, as well as drugs (Han et al., 2016; Piccinin et al., 2019). Metabolomics can help us to comprehensively analyze the alterations of key metabolites in disease and physiological processes, predict biomarkers of disease, and find active drivers of biological processes (Rinschen et al., 2019). In our study, the results of hepatic metabolomics showed a considerable drop in glutathione and glucose 6-phosphate levels, after 8 weeks of PM2.5 exposure. Among them, glutathione plays a significant role in the TCA cycle and can promote lipid metabolism (Gao et al., 2021). In addition, there was a significant increase in stearidonic acid, which is an omega-3 polyunsaturated fatty acid that affects steroid synthesis and alters the lipid profile (Shahidi and Ambigaipalan, 2018).

Transcriptome analysis in the liver was performed to investigate the altered signaling pathways in order to further study the underlying molecular mechanism. Overall, the metabolic pathways were found significant upregulated after exposed to PM2.5, which was consistent with the metabolomic results. Our results showed that fatty acid metabolism, fatty acid degradation, fatty acid elongation, biosynthesis of unsaturated fatty acids, oxidative phosphorylation pathways are significantly altered in the liver after 8 weeks of PM2.5 exposure. These signaling pathway are involved in regulating the balance of lipid and fatty acid, including Tiglylglycine, Stearidonic acid, (10E,12Z)−octadecadienoic acid and so on. Moreover, according to transcriptome analysis the Glycolysis/Gluconeogenesis pathway was upregulated in PM group, consistent with the alteration of Glucose-6-phosphate in metabolomics analysis. Subsequently, the genes related to fatty acid synthesis and oxidation were validated by qPCR. After 8 weeks of exposure, the expression level of *PPARα*, a key factor controlling fatty acid oxidation, was enhanced, and the expression levels of the DNL-related genes *FAS* and *SCD1* were decreased. Plasma levels of VLDL increased significantly after 8 weeks of exposure. Similarly, the mRNA levels of FAS and *SCD1* in gWAT and sWAT increased markedly, and *ACC1* also increased markedly in sWAT.

Mitochondria are considered the main sites of fatty acid oxidation and serve as the “powerhouse” for energy production. PM2.5 in ambient air induces mitochondrial damage in the mouse organism, including liver, heart and other tissues (Qiu et al., 2019; Jiang et al., 2021). Mitochondrial damage and lipid metabolism disorders promote each other. In detail, when mitochondrial dysfunction affects the homeostasis of lipid metabolism, increased tissue lipid supply and incomplete lipid oxidation will act back on the mitochondria (Gonzalez-Franquesa and Patti, 2017; Sangwung et al., 2020). Considering the central role in metabolism, mitochondrial activity was examined in our study (Xu et al., 2011; Mansouri et al., 2018). The main pathway of fat degradation is mitochondrial FAO (Houten et al., 2016). *CPT1* and *CPT2* are located in the inner and outer mitochondrial membranes, respectively. *CPT1* and *CPT2* transfer fatty acids into the mitochondria for further processing and play an integral role in the mitochondrial FAO process (Nassir and Ibdah, 2014). *PGC1α*, an important regulator of mitochondrial proliferation, was reported to expand the mitochondrial number and mitochondrial oxidative activity by boosting *CPT1* activity (Villena, 2015; Jamwal et al., 2021). The mRNA and protein expression levels of *CPT1*, *CPT2*, and *PGC1α* were substantially higher in the liver and adipose tissue after PM2.5 exposure.

However, our study was conducted in winter time to ensure exposure to high PM2.5 concentration, resulting in relative short-term exposure. Moreover, we applied young female mice, which could not observe the gender difference under air pollution. In addition, we only examined the external exposure dose, but the internal exposure dose was not evaluated in the study.

## 5 Conclusion

In summary, our study reported the hypertrophy of adipose tissue and revealed a comprehensive analysis of lipidomic and metabolic profiles combined with transcriptomics analysis in young mice under real-ambient exposure to PM2.5. The real-ambient exposure system provided a perfect model to study the hazardous effects of air pollution due to similar concentrations and chemical characteristics of PM2.5 between the exposure system and outdoor air. The differences in metabolic compositions reflected the imbalance in lipid metabolism. Taken together, our research investigated the hypotrophy of adipose tissue in young mice, supported an imbalance in lipid metabolism based on multi-omics analysis, and revealed disordered mitochondrial function under PM2.5 exposure. The present study further extends our understanding about the health effects of exposure to PM2.5 at an early age and provide evidence-based support for epidemiological studies on air pollution increasing the prevalence of overweight or obesity in children and adolescents.

## Data Availability

Original datasets are available in a publicly accessible repository: The original contributions presented in the study are publicly available. This data can be found here: www.ebi.ac.uk/metabolights/MTBLS7396, www.ebi.ac.uk/metabolights/MTBLS7397.
