# Supplementary material for: Multi-omics reveals hypertrophy of adipose tissue and lipid metabolism disorder via mitochondria in young mice under real-ambient exposure to air pollution
Source: Front Pharmacol. 2023 Mar 23;14:1122615. doi: 10.3389/fphar.2023.1122615 (PMC10079078; doi:10.3389/fphar.2023.1122615)
Supplement: Supplementary file 1 [file Table1.docx]

**Multi-omics reveals hypertrophy of adipose tissue and lipid metabolism disorder via mitochondria in young mice under real-ambient exposure to air pollution**

**Honglin Si^1^**^✝^**, Tianlin Gao^1✝^, Jing Yang^2^, Jing Zhu^2^, Ying Han^2^, Chengwei Li^3^, Jianxin Wang^1^, Jianyu Li^1^, Yanjie Zhao^1^, Lei Chen^1^, Yuxin Zheng^1^, Menghui Jiang^1,*^**

^1^School of Public Health, Qingdao University, Qingdao, China

^2^School of Life Science and Technology, ShanghaiTech University, Shanghai, China

^3^Linyi Center for Disease Control and Prevention, Linyi, China

* Correspondence: jiangmenghui@qdu.edu.cn

✝ These authors contributed equally to this work and share first authorship

**Supplementary Table 1.** **Primer sequences**

| Mouse ACC1 | F: GATGAACCATCTCCGTTGGC  R: GACCCAATTATGAATCGGGAGTG |
| --- | --- |
| Mouse ACC2 | F: ACCAGCTTTATCCTGGGCTC  R: GACGGTGAAATCTCTGTGCAG |
| Mouse FAS | F: GGCTCTATGGATTACCCAAGC  R: CCAGTGTTCGTTCCTCGGA |
| Mouse SCD1 | F: TTCTTGCGATACACTCTGGTGC  R: CGGGATTGAATGTTCTTGTCGT |
| Mouse Srebp1c | F: GATGTGCGAACTGGACACAG  R: CATAGGGGGCGTCAAACAG |
| Mouse PPARα | F: AACATCGAGTGTCGAATATGTGG  R: CCGAATAGTTCGCCGAAAGAA |
| Mouse Hadh | F: CCTGGATTCATCGTGAACCGA  R: GATGTCTTCCTTAGACGCATCG |
| Mouse Hadha | F: GAGCTTTCGTCCTCTTCTGCT  R: CTGAAGGCACTCCGAGACT |
| Mouse Hadhb | F: TGAATATGCACTGCGTTCTCAT  R: CCTTTCCTGGTACTTTGAAGGG |
| Mouse Ehhadh | F: GTTTGGACCATACGGTTAGAGC  R: GCTTCTGGTATCGCTGTATTTCA |
| Mouse Acads | F: GACTGGCGACGGTTACACA  R: GGCAAAGTCACGGCATGTC |
| Mouse Acadl | F: TGCCCTATATTGCGAATTACGG  R: CTATGGCACCGATACACTTGC |
| Mouse DLOOP | F: AATCTACCATCCTCCGTG  R: GACTAATGATTCTTCACCGT |
| Mouse 18S | F: CATTCGAACGTCTGCCCTATC  R: CCTGCTGCCTTCCTTGGA |
| Mouse CPT1 | F: TGGCATCATCACTGGTGTGTT  R: GTCTAGGGTCCGATTGATCTTTG |
| Mouse CPT2 | F: CAGCACAGCATCGTACCCA  R: TCCCAATGCCGTTCTCAAAAT |
| Mouse PGC1α | F: TATGGAGTGACATAGAGTGTGCT  R: GTCGCTACACCACTTCAATCC |
